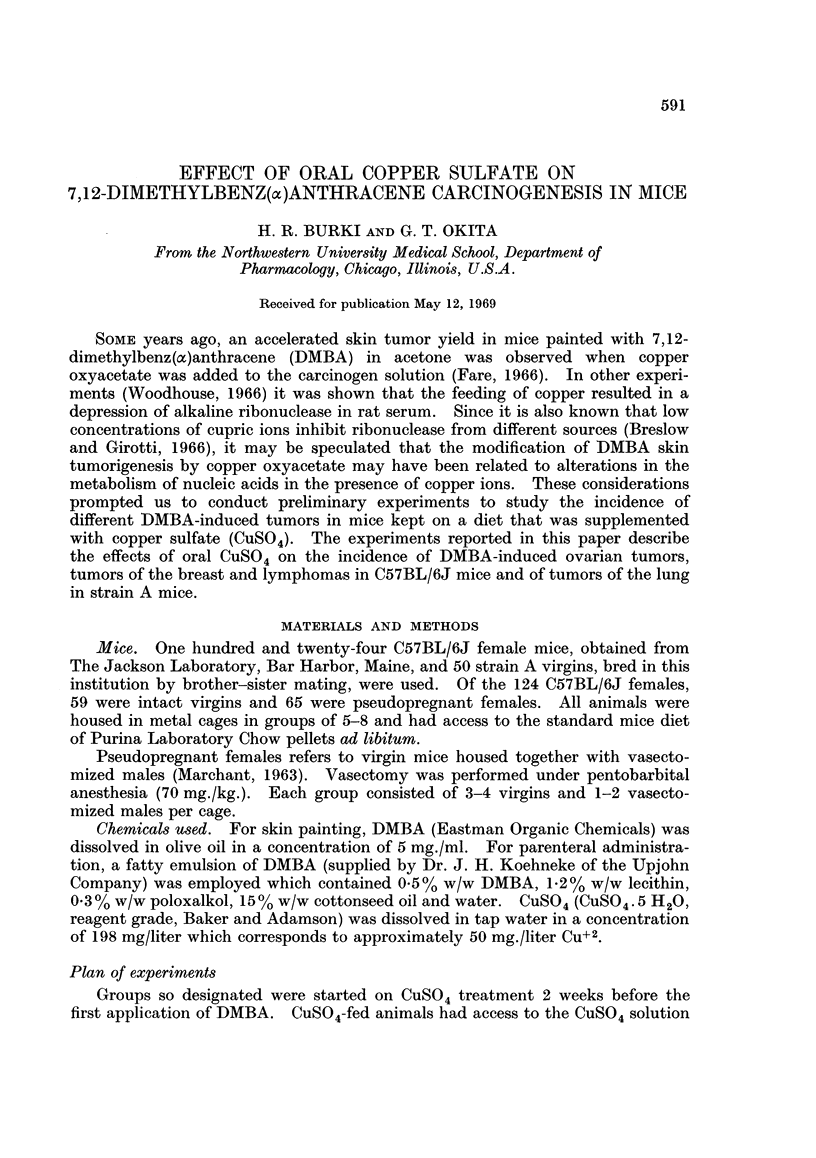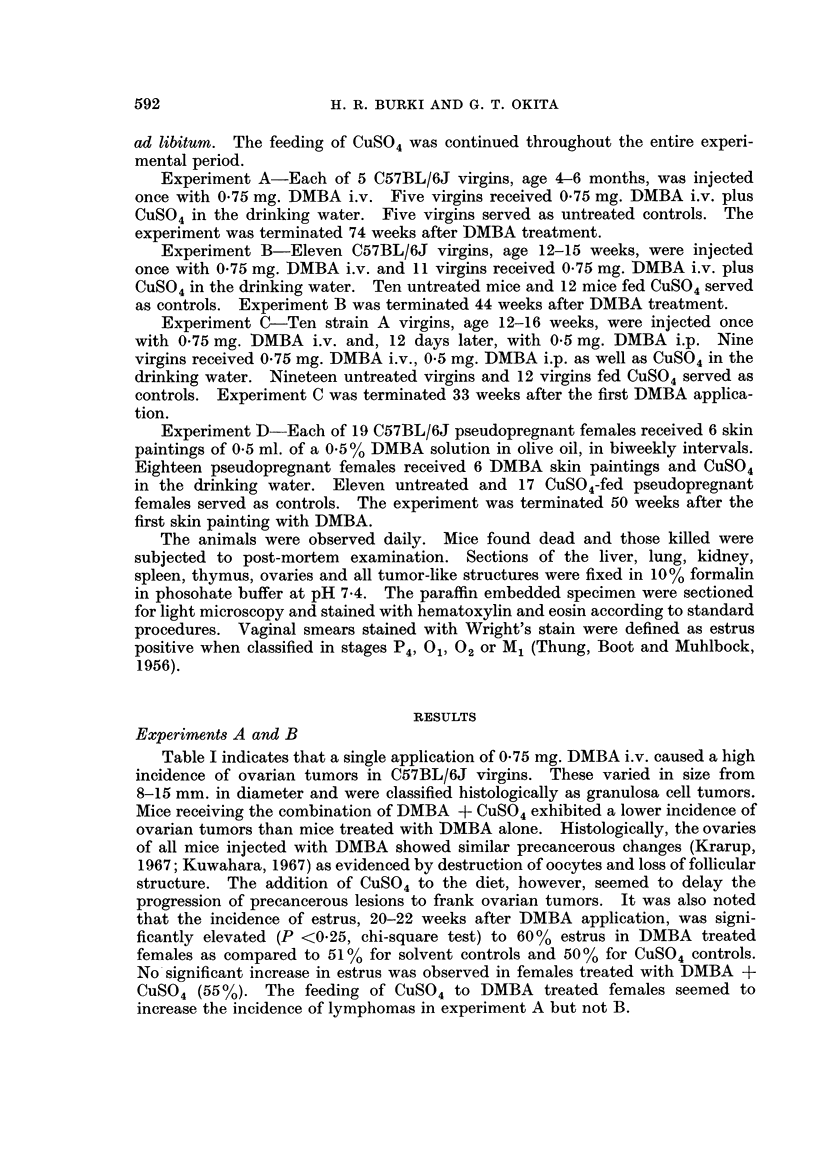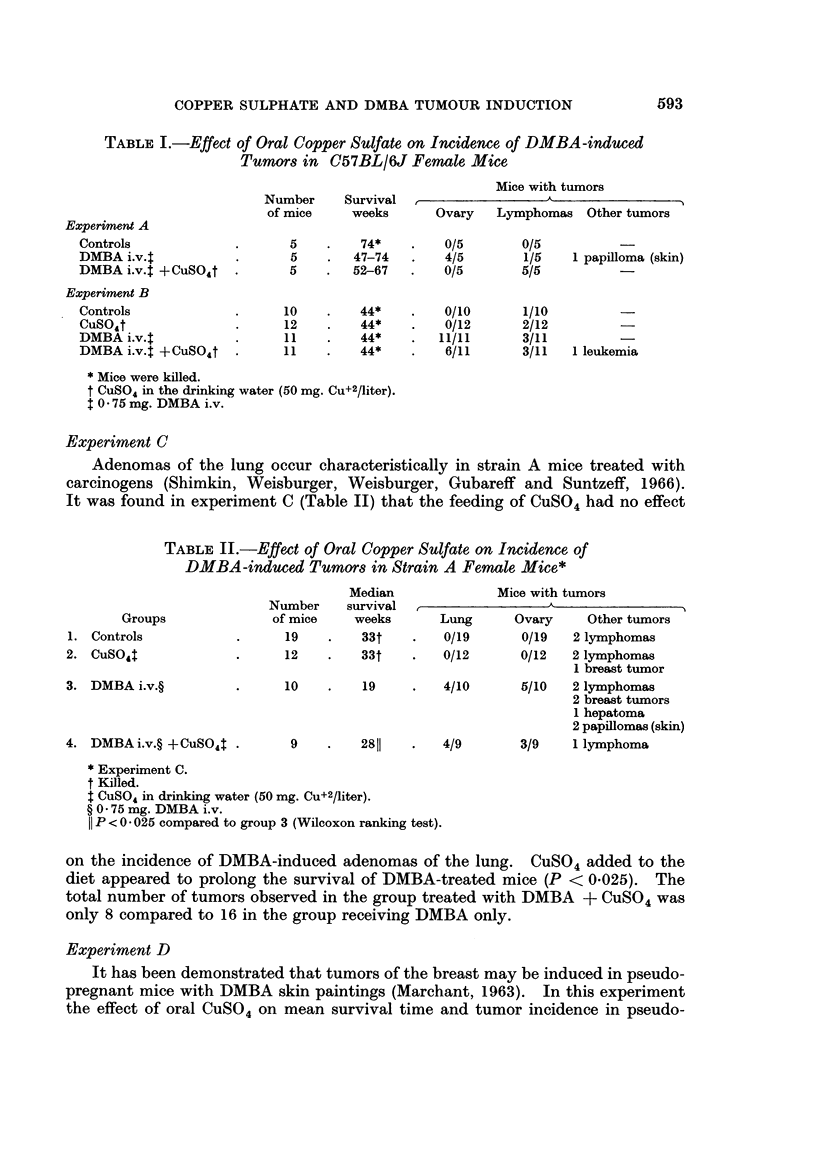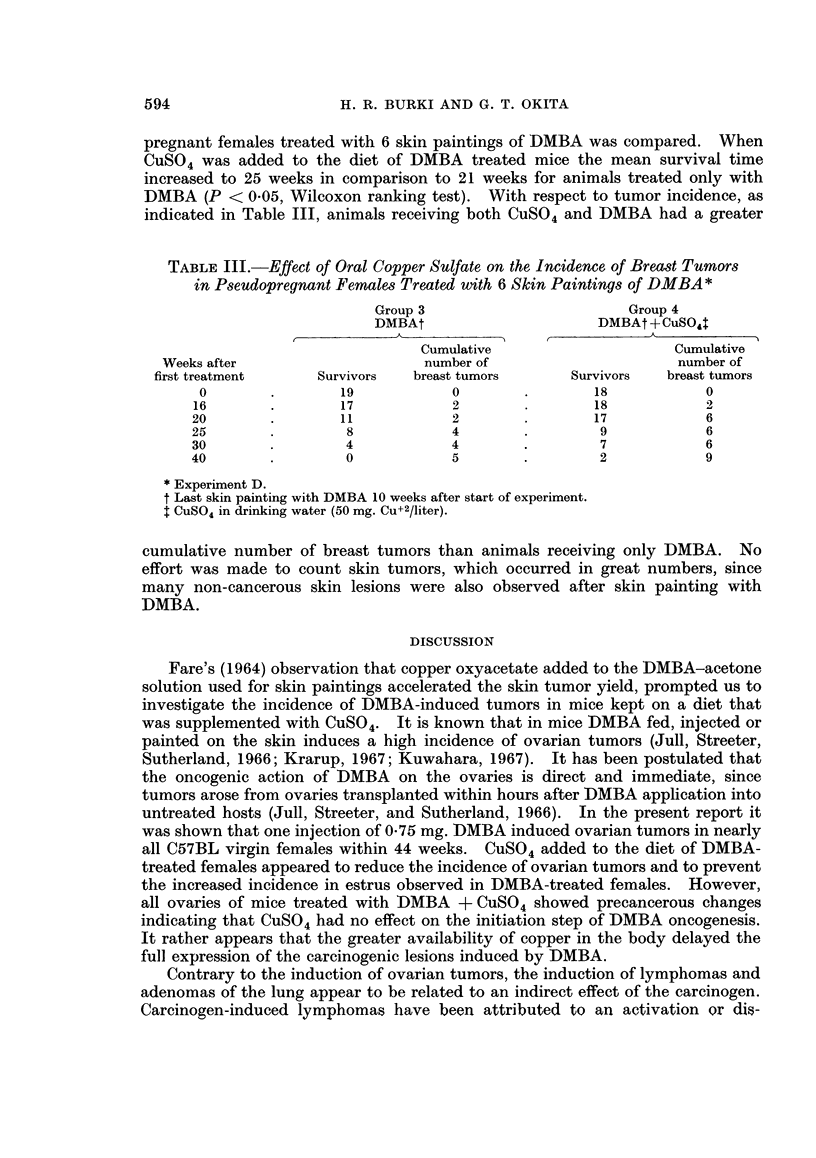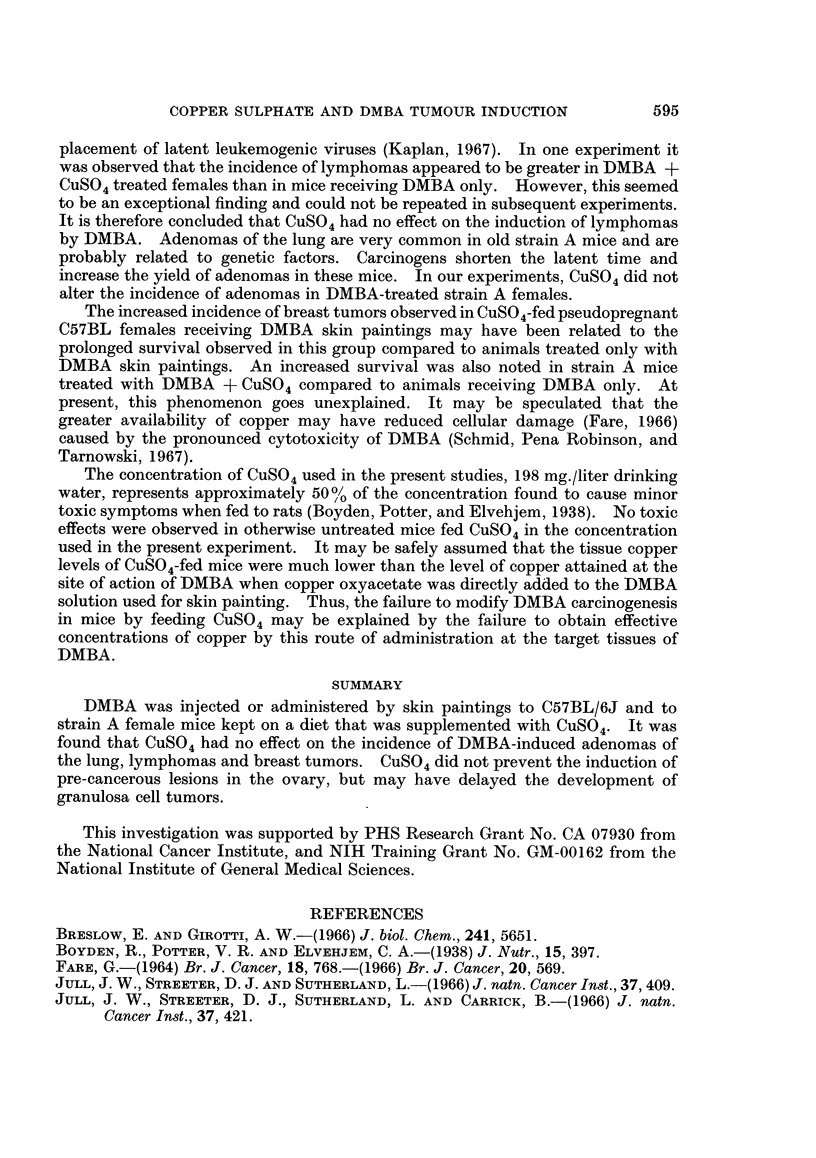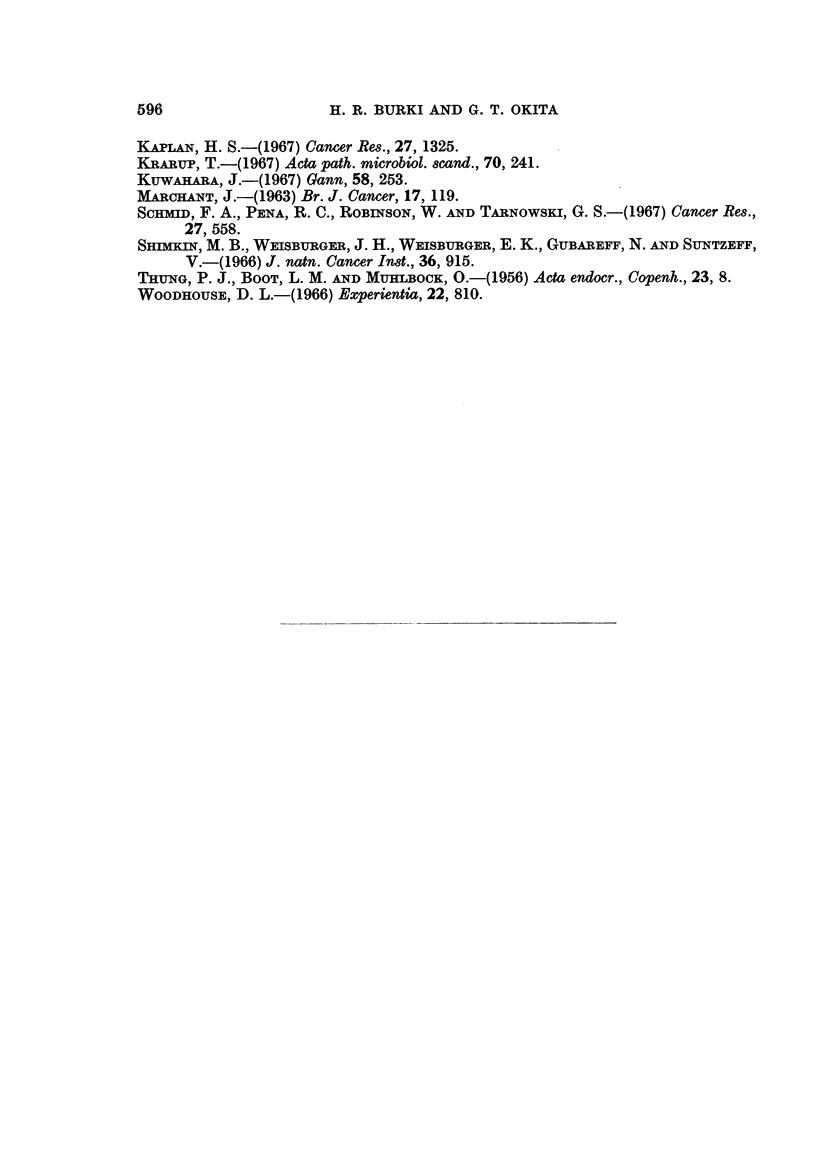# Effect of oral copper sulfate on 7,12-dimethylbenz(alpha)anthracene carcinogenesis in mice.

**DOI:** 10.1038/bjc.1969.73

**Published:** 1969-09

**Authors:** H. R. Burki, G. T. Okita


### EFFECT OF ORAL COPPER SULFATE ON 7,12-DIMETHYLBENZ(α)ANTHRACENE CARCINOGENESIS IN MICE

H. R. BURKI AND G. T. OKITA

*From the Northwestern University Medical School, Department of Pharmacology, Chicago, Illinois, U.S.A.*

Received for publication May 12, 1969

SOME years ago, an accelerated skin tumor yield in mice painted with 7,12-dimethylbenz(α)anthracene (DMBA) in acetone was observed when copper oxyacetate was added to the carcinogen solution (Fare, 1966). In other experiments (Woodhouse, 1966) it was shown that the feeding of copper resulted in a depression of alkaline ribonuclease in rat serum. Since it is also known that low concentrations of cupric ions inhibit ribonuclease from different sources (Breslow and Girotti, 1966), it may be speculated that the modification of DMBA skin tumorigenesis by copper oxyacetate may have been related to alterations in the metabolism of nucleic acids in the presence of copper ions. These considerations prompted us to conduct preliminary experiments to study the incidence of different DMBA-induced tumors in mice kept on a diet that was supplemented with copper sulfate ($CuSO_4$). The experiments reported in this paper describe the effects of oral $CuSO_4$ on the incidence of DMBA-induced ovarian tumors, tumors of the breast and lymphomas in C57BL/6J mice and of tumors of the lung in strain A mice.

### MATERIALS AND METHODS

*Mice.* One hundred and twenty-four C57BL/6J female mice, obtained from The Jackson Laboratory, Bar Harbor, Maine, and 50 strain A virgins, bred in this institution by brother–sister mating, were used. Of the 124 C57BL/6J females, 59 were intact virgins and 65 were pseudopregnant females. All animals were housed in metal cages in groups of 5–8 and had access to the standard mice diet of Purina Laboratory Chow pellets *ad libitum.*

Pseudopregnant females refers to virgin mice housed together with vasectomized males (Marchant, 1963). Vasectomy was performed under pentobarbital anesthesia (70 mg./kg.). Each group consisted of 3–4 virgins and 1–2 vasectomized males per cage.

*Chemicals used.* For skin painting, DMBA (Eastman Organic Chemicals) was dissolved in olive oil in a concentration of 5 mg./ml. For parenteral administration, a fatty emulsion of DMBA (supplied by Dr. J. H. Koehneke of the Upjohn Company) was employed which contained 0·5% w/w DMBA, 1·2% w/w lecithin, 0·3% w/w poloxalkol, 15% w/w cottonseed oil and water. $CuSO_4$ ($CuSO_4 . 5\ H_2O$, reagent grade, Baker and Adamson) was dissolved in tap water in a concentration of 198 mg/liter which corresponds to approximately 50 mg./liter $Cu^{+2}$.

### *Plan of experiments*

Groups so designated were started on $CuSO_4$ treatment 2 weeks before the first application of DMBA. $CuSO_4$-fed animals had access to the $CuSO_4$ solution

*ad libitum*. The feeding of $CuSO_4$ was continued throughout the entire experimental period.

Experiment A—Each of 5 C57BL/6J virgins, age 4–6 months, was injected once with 0·75 mg. DMBA i.v. Five virgins received 0·75 mg. DMBA i.v. plus $CuSO_4$ in the drinking water. Five virgins served as untreated controls. The experiment was terminated 74 weeks after DMBA treatment.

Experiment B—Eleven C57BL/6J virgins, age 12–15 weeks, were injected once with 0·75 mg. DMBA i.v. and 11 virgins received 0·75 mg. DMBA i.v. plus $CuSO_4$ in the drinking water. Ten untreated mice and 12 mice fed $CuSO_4$ served as controls. Experiment B was terminated 44 weeks after DMBA treatment.

Experiment C—Ten strain A virgins, age 12–16 weeks, were injected once with 0·75 mg. DMBA i.v. and, 12 days later, with 0·5 mg. DMBA i.p. Nine virgins received 0·75 mg. DMBA i.v., 0·5 mg. DMBA i.p. as well as $CuSO_4$ in the drinking water. Nineteen untreated virgins and 12 virgins fed $CuSO_4$ served as controls. Experiment C was terminated 33 weeks after the first DMBA application.

Experiment D—Each of 19 C57BL/6J pseudopregnant females received 6 skin paintings of 0·5 ml. of a 0·5% DMBA solution in olive oil, in biweekly intervals. Eighteen pseudopregnant females received 6 DMBA skin paintings and $CuSO_4$ in the drinking water. Eleven untreated and 17 $CuSO_4$-fed pseudopregnant females served as controls. The experiment was terminated 50 weeks after the first skin painting with DMBA.

The animals were observed daily. Mice found dead and those killed were subjected to post-mortem examination. Sections of the liver, lung, kidney, spleen, thymus, ovaries and all tumor-like structures were fixed in 10% formalin in phosohate buffer at pH 7·4. The paraffin embedded specimen were sectioned for light microscopy and stained with hematoxylin and eosin according to standard procedures. Vaginal smears stained with Wright's stain were defined as estrus positive when classified in stages $P_4$, $O_1$, $O_2$ or $M_1$ (Thung, Boot and Muhlbock, 1956).

### RESULTS

### *Experiments A and B*

Table I indicates that a single application of 0·75 mg. DMBA i.v. caused a high incidence of ovarian tumors in C57BL/6J virgins. These varied in size from 8–15 mm. in diameter and were classified histologically as granulosa cell tumors. Mice receiving the combination of DMBA + $CuSO_4$ exhibited a lower incidence of ovarian tumors than mice treated with DMBA alone. Histologically, the ovaries of all mice injected with DMBA showed similar precancerous changes (Krarup, 1967; Kuwahara, 1967) as evidenced by destruction of oocytes and loss of follicular structure. The addition of $CuSO_4$ to the diet, however, seemed to delay the progression of precancerous lesions to frank ovarian tumors. It was also noted that the incidence of estrus, 20–22 weeks after DMBA application, was significantly elevated ($P < 0·25$, chi-square test) to 60% estrus in DMBA treated females as compared to 51% for solvent controls and 50% for $CuSO_4$ controls. No significant increase in estrus was observed in females treated with DMBA + $CuSO_4$ (55%). The feeding of $CuSO_4$ to DMBA treated females seemed to increase the incidence of lymphomas in experiment A but not B.

TABLE I.—*Effect of Oral Copper Sulfate on Incidence of DMBA-induced Tumors in C57BL/6J Female Mice*

| | Number of mice | Survival weeks | Mice with tumors: Ovary | Lymphomas | Other tumors |
|---|---|---|---|---|---|
| *Experiment A* | | | | | |
| Controls | 5 | 74* | 0/5 | 0/5 | — |
| DMBA i.v.‡ | 5 | 47–74 | 4/5 | 1/5 | 1 papilloma (skin) |
| DMBA i.v.‡ + $CuSO_4$† | 5 | 52–67 | 0/5 | 5/5 | — |
| *Experiment B* | | | | | |
| Controls | 10 | 44* | 0/10 | 1/10 | — |
| $CuSO_4$† | 12 | 44* | 0/12 | 2/12 | — |
| DMBA i.v.‡ | 11 | 44* | 11/11 | 3/11 | — |
| DMBA i.v.‡ + $CuSO_4$† | 11 | 44* | 6/11 | 3/11 | 1 leukemia |

* Mice were killed.
† $CuSO_4$ in the drinking water (50 mg. $Cu^{+2}$/liter).
‡ 0·75 mg. DMBA i.v.

*Experiment C*

Adenomas of the lung occur characteristically in strain A mice treated with carcinogens (Shimkin, Weisburger, Weisburger, Gubareff and Suntzeff, 1966). It was found in experiment C (Table II) that the feeding of $CuSO_4$ had no effect

TABLE II.—*Effect of Oral Copper Sulfate on Incidence of DMBA-induced Tumors in Strain A Female Mice**

| Groups | Number of mice | Median survival weeks | Mice with tumors: Lung | Ovary | Other tumors |
|---|---|---|---|---|---|
| 1. Controls | 19 | 33† | 0/19 | 0/19 | 2 lymphomas |
| 2. $CuSO_4$‡ | 12 | 33† | 0/12 | 0/12 | 2 lymphomas<br>1 breast tumor |
| 3. DMBA i.v.§ | 10 | 19 | 4/10 | 5/10 | 2 lymphomas<br>2 breast tumors<br>1 hepatoma<br>2 papillomas (skin) |
| 4. DMBA i.v.§ + $CuSO_4$‡ | 9 | 28‖ | 4/9 | 3/9 | 1 lymphoma |

* Experiment C.
† Killed.
‡ $CuSO_4$ in drinking water (50 mg. $Cu^{+2}$/liter).
§ 0·75 mg. DMBA i.v.
‖ $P < 0{\cdot}025$ compared to group 3 (Wilcoxon ranking test).

on the incidence of DMBA-induced adenomas of the lung. $CuSO_4$ added to the diet appeared to prolong the survival of DMBA-treated mice ($P < 0{\cdot}025$). The total number of tumors observed in the group treated with DMBA + $CuSO_4$ was only 8 compared to 16 in the group receiving DMBA only.

*Experiment D*

It has been demonstrated that tumors of the breast may be induced in pseudo-pregnant mice with DMBA skin paintings (Marchant, 1963). In this experiment the effect of oral $CuSO_4$ on mean survival time and tumor incidence in pseudopregnant females treated with 6 skin paintings of DMBA was compared. When $CuSO_4$ was added to the diet of DMBA treated mice the mean survival time increased to 25 weeks in comparison to 21 weeks for animals treated only with DMBA ($P < 0{\cdot}05$, Wilcoxon ranking test). With respect to tumor incidence, as indicated in Table III, animals receiving both $CuSO_4$ and DMBA had a greater

TABLE III.—*Effect of Oral Copper Sulfate on the Incidence of Breast Tumors in Pseudopregnant Females Treated with* 6 *Skin Paintings of DMBA**

| | Group 3 DMBA† | | Group 4 DMBA† + $CuSO_4$‡ | |
|---|---|---|---|---|
| Weeks after first treatment | Survivors | Cumulative number of breast tumors | Survivors | Cumulative number of breast tumors |
| 0 | 19 | 0 | 18 | 0 |
| 16 | 17 | 2 | 18 | 2 |
| 20 | 11 | 2 | 17 | 6 |
| 25 | 8 | 4 | 9 | 6 |
| 30 | 4 | 4 | 7 | 6 |
| 40 | 0 | 5 | 2 | 9 |

* Experiment D.
† Last skin painting with DMBA 10 weeks after start of experiment.
‡ $CuSO_4$ in drinking water (50 mg. $Cu^{+2}$/liter).

cumulative number of breast tumors than animals receiving only DMBA. No effort was made to count skin tumors, which occurred in great numbers, since many non-cancerous skin lesions were also observed after skin painting with DMBA.

### DISCUSSION

Fare's (1964) observation that copper oxyacetate added to the DMBA–acetone solution used for skin paintings accelerated the skin tumor yield, prompted us to investigate the incidence of DMBA-induced tumors in mice kept on a diet that was supplemented with $CuSO_4$. It is known that in mice DMBA fed, injected or painted on the skin induces a high incidence of ovarian tumors (Jull, Streeter, Sutherland, 1966; Krarup, 1967; Kuwahara, 1967). It has been postulated that the oncogenic action of DMBA on the ovaries is direct and immediate, since tumors arose from ovaries transplanted within hours after DMBA application into untreated hosts (Jull, Streeter, and Sutherland, 1966). In the present report it was shown that one injection of 0·75 mg. DMBA induced ovarian tumors in nearly all C57BL virgin females within 44 weeks. $CuSO_4$ added to the diet of DMBA-treated females appeared to reduce the incidence of ovarian tumors and to prevent the increased incidence in estrus observed in DMBA-treated females. However, all ovaries of mice treated with DMBA + $CuSO_4$ showed precancerous changes indicating that $CuSO_4$ had no effect on the initiation step of DMBA oncogenesis. It rather appears that the greater availability of copper in the body delayed the full expression of the carcinogenic lesions induced by DMBA.

Contrary to the induction of ovarian tumors, the induction of lymphomas and adenomas of the lung appear to be related to an indirect effect of the carcinogen. Carcinogen-induced lymphomas have been attributed to an activation or displacement of latent leukemogenic viruses (Kaplan, 1967). In one experiment it was observed that the incidence of lymphomas appeared to be greater in DMBA + $CuSO_4$ treated females than in mice receiving DMBA only. However, this seemed to be an exceptional finding and could not be repeated in subsequent experiments. It is therefore concluded that $CuSO_4$ had no effect on the induction of lymphomas by DMBA. Adenomas of the lung are very common in old strain A mice and are probably related to genetic factors. Carcinogens shorten the latent time and increase the yield of adenomas in these mice. In our experiments, $CuSO_4$ did not alter the incidence of adenomas in DMBA-treated strain A females.

The increased incidence of breast tumors observed in $CuSO_4$-fed pseudopregnant C57BL females receiving DMBA skin paintings may have been related to the prolonged survival observed in this group compared to animals treated only with DMBA skin paintings. An increased survival was also noted in strain A mice treated with DMBA + $CuSO_4$ compared to animals receiving DMBA only. At present, this phenomenon goes unexplained. It may be speculated that the greater availability of copper may have reduced cellular damage (Fare, 1966) caused by the pronounced cytotoxicity of DMBA (Schmid, Pena Robinson, and Tarnowski, 1967).

The concentration of $CuSO_4$ used in the present studies, 198 mg./liter drinking water, represents approximately 50% of the concentration found to cause minor toxic symptoms when fed to rats (Boyden, Potter, and Elvehjem, 1938). No toxic effects were observed in otherwise untreated mice fed $CuSO_4$ in the concentration used in the present experiment. It may be safely assumed that the tissue copper levels of $CuSO_4$-fed mice were much lower than the level of copper attained at the site of action of DMBA when copper oxyacetate was directly added to the DMBA solution used for skin painting. Thus, the failure to modify DMBA carcinogenesis in mice by feeding $CuSO_4$ may be explained by the failure to obtain effective concentrations of copper by this route of administration at the target tissues of DMBA.

### SUMMARY

DMBA was injected or administered by skin paintings to C57BL/6J and to strain A female mice kept on a diet that was supplemented with $CuSO_4$. It was found that $CuSO_4$ had no effect on the incidence of DMBA-induced adenomas of the lung, lymphomas and breast tumors. $CuSO_4$ did not prevent the induction of pre-cancerous lesions in the ovary, but may have delayed the development of granulosa cell tumors.

This investigation was supported by PHS Research Grant No. CA 07930 from the National Cancer Institute, and NIH Training Grant No. GM-00162 from the National Institute of General Medical Sciences.

### REFERENCES

BRESLOW, E. AND GIROTTI, A. W.—(1966) *J. biol. Chem.*, **241**, 5651.
BOYDEN, R., POTTER, V. R. AND ELVEHJEM, C. A.—(1938) *J. Nutr.*, **15**, 397.
FARE, G.—(1964) *Br. J. Cancer*, **18**, 768.—(1966) *Br. J. Cancer*, **20**, 569.
JULL, J. W., STREETER, D. J. AND SUTHERLAND, L.—(1966) *J. natn. Cancer Inst.*, **37**, 409.
JULL, J. W., STREETER, D. J., SUTHERLAND, L. AND CARRICK, B.—(1966) *J. natn. Cancer Inst.*, **37**, 421.

KAPLAN, H. S.—(1967) *Cancer Res.*, **27**, 1325.
KRARUP, T.—(1967) *Acta path. microbiol. scand.*, **70**, 241.
KUWAHARA, J.—(1967) *Gann*, **58**, 253.
MARCHANT, J.—(1963) *Br. J. Cancer*, **17**, 119.
SCHMID, F. A., PENA, R. C., ROBINSON, W. AND TARNOWSKI, G. S.—(1967) *Cancer Res.*, **27**, 558.
SHIMKIN, M. B., WEISBURGER, J. H., WEISBURGER, E. K., GUBAREFF, N. AND SUNTZEFF, V.—(1966) *J. natn. Cancer Inst.*, **36**, 915.
THUNG, P. J., BOOT, L. M. AND MUHLBOCK, O.—(1956) *Acta endocr., Copenh.*, **23**, 8.
WOODHOUSE, D. L.—(1966) *Experientia*, **22**, 810.